# Clinical and cost effectiveness of staff training in Positive Behaviour Support (PBS) for treating challenging behaviour in adults with intellectual disability: a cluster randomised controlled trial

**DOI:** 10.1186/s12888-014-0219-6

**Published:** 2014-08-03

**Authors:** Angela Hassiotis, Andre Strydom, Mike Crawford, Ian Hall, Rumana Omar, Victoria Vickerstaff, Rachael Hunter, Jason Crabtree, Vivien Cooper, Asit Biswas, William Howie, Michael King

**Affiliations:** UCL Division of Psychiatry, Charles Bell House, 67-73 Riding House Street, London, W1W 7EY UK; Imperial College of Medicine, Claybrook Centre, Charing Cross Campus, 37 Claybrook Road, London, W6 8LN UK; East London Foundation Trust, Community Learning Disability Service, Mile End Hospital, Bancroft Road, London, UK; Medical Statistics, Department of Statistical Science, University College London, Gower Street, London, WC1E 6BT UK; The Research Department of Primary Care and Population Sciences, University College, Rowland Hill Street, London, NW3 2PF UK; University College London, Primary Care & Population Health, Royal Free Hospital, Rowland Street, London, NW3 2PF UK; Department of Clinical Psychology, University College London, 119 Torrington Place, London, WC1E 7HB UK; Challenging Behaviour Foundation, The Old Courthouse, New Road Avenue, Chatham, ME4 6BE UK; Leicestershire Partnership NHS Trust, Directorate of Learning Disabilities, Frith Hospital, Groby Road, Leicester, LE3 9QF UK; South West London and St George’s Mental Health Trust, Wandsworth Community Mental Health Learning Disabilities Team, Joan Bicknell Centre, Springfield Hospital, London, SW17 7DJ UK

**Keywords:** Challenging behaviour, Cluster RCT, Positive Behaviour Support, Intellectual disability, Complex intervention, Behavioural intervention

## Abstract

**Background:**

Many people with intellectual disability present with challenging behaviour which often has serious consequences such as the prescription of long term medication, in-patient admissions and disruption of normal daily activities. Small scale studies of Positive Behaviour Support (PBS) delivered by paid carers suggest that it reduces challenging behaviour and costs of care and improves quality of life. This study aims to investigate whether professionals training in the delivery of PBS as part of routine practice is clinically and cost effective compared to treatment as usual in community intellectual disability services.

**Method:**

The study is a multi-centre cluster randomised controlled trial involving community intellectual disability services in England and service users with mild to severe intellectual disability and challenging behaviour. The teams will be randomly allocated into one of two conditions, either training and support to deliver PBS or treatment as usual. We will carry out assessments of challenging behaviour, use of services, quality of life, mental health, and family and paid carer burden at six and 12 months. We will monitor treatment fidelity and we will interview a sample of paid and family carers, service users, staff and managers about what they think of the treatment and how best we can deliver it in routine care. The main outcome is reduction in challenging behaviour at one year after randomisation. We will also carry out a health economic evaluation to examine the costs and consequences of staff training in PBS.

**Discussion:**

The study findings will have significant implications for the delivery of PBS in community based services with the potential for reducing inpatient admissions and out-of-area placements for adults with intellectual disability and challenging behaviour.

**Trial registration:**

This trial is registered with Clinical Trials.gov (Ref NCT01680276).

Clinical Trials Unit: PRIMENT https://www.ucl.ac.uk/priment/.

**Electronic supplementary material:**

The online version of this article (doi:10.1186/s12888-014-0219-6) contains supplementary material, which is available to authorized users.

## Background

Intellectual disability is a disorder that includes impairments in three domains, i.e. cognitive ability, social skills and adaptive abilities and it should be evident during development [1]. There are estimated to be 800,000 people with mild and 112,000 people with severe intellectual disability in the UK [2]. Challenging behaviours are common in this population with a point prevalence in adults of 22.5% and a two year incidence of 4.6% [3]. Aggression is by far the commonest type of challenging behaviour with a reported prevalence of 11% to 27% [4]. Challenging behaviour is associated with increase in receipt of antipsychotic medication, service use, hospitalisation, restrictive care practices and deprivation [5-8]

Reduction of challenging behaviour has been the focus of many interventional approaches over the years. Most of the literature comprises small-n and single case empirical research delivering a variety of pharmacological and psychosocial treatments [9-12]. However, there are few well conducted RCTs and the findings of those that have been carried out are inconclusive; most notably, a trial of antipsychotics showed that they were no more effective than placebo [13] in reducing challenging behaviour. Overall, existing RCTs are small, subject to bias, have only examined short-term outcomes and lack a cost effectiveness component or reports on health related quality of life. A pilot RCT (n = 63) showed that Applied Behaviour Analysis (ABA) delivered by a specialist team can significantly improve irritability, lethargy and hyperactivity and may be cost neutral over six months [11]. Furthermore, a naturalistic follow-up for two years after randomisation showed a sustained positive effect of the intervention on challenging behaviour [12]. An additional qualitative exploration of the opinions of those involved in the trial (patients, carers and staff) revealed that there was strong support for clinical trials as a method of evaluating psychological therapies to treat challenging behaviour [14].

### The financial burden of challenging behaviour

European data on the cost of disorders of the brain [15] estimate that the cost of intellectual disabilities is 43bn euros most of which is accounted for by costs of direct health and non-medical care. A survey of five London areas showed that 67% (£14 million, 2004–2005 prices) of combined medical and social care budgets supported 134 out-of-area placements for adults with intellectual disability and challenging behaviour [16]. Individual care packages commonly ranged from £100 k to 450 k per annum. Other studies indicate that costs are higher for people with more severe intellectual disability and those with greater levels of challenging behaviour, with specialist accommodation accounting for the majority of the cost [17-19].

### The origins of PBS

Positive Behaviour Support (PBS) and its precursor ABA are multicomponent methods that purport to support people with intellectual disability and challenging behaviour [20,21]. PBS more specifically has developed into a discipline that focuses on the influence of the environment on behaviour rather than solely on the internal drivers of the behaviours [22]. PBS based practice consists of a flexible, multicomponent intervention which takes a lifespan perspective, emphasises prevention and fosters stakeholder participation. PBS practice includes a functional assessment of the possible relationships between specific environmental events and the target behaviours. By identifying what reinforces the behaviour, practitioners can put in place interventions which are designed to foster prosocial actions. The final aim is to enhance the person’s quality of life and his/her integration within the local community. An important tenet of PBS is that challenging behaviour is shaped by personal and psychological experiences and helps the person, with often severe intellectual disability, to exert some control over his/her environment. The challenging behaviour may be a response to environmental cues or “schedule-induced”, that is, present as a result of interactions between the individual and the environment. These concepts have influenced the definition of challenging behaviour as “*behaviour of such intensity, frequency or duration that the physical safety of the person or others is placed in serious jeopardy or behaviour which is likely to seriously limit or deny access to the use of ordinary community facilities*” [23].

PBS is a widely applied complex intervention to a number of conditions such as autism spectrum disorders, in education, mental health and other fields in health and social care.

### PBS based staff training

Specific staff competencies are a crucial factor in maintaining improvements in behaviour. However, given the resource associated with delivering PBS, only about half of service users in need of PBS may be treated at any time [24]. Lack of competencies in managing challenging behaviour is also an important factor in perpetuating out-of-area placements [25,26]. Even where there are tertiary specialist teams which provide ABA/PBS, service users often have to wait several months to receive help.

One approach to meeting this shortfall is to train paid carers and professional staff in PBS. McClean et al. [24] and Grey & McClean [27] have reported descriptive data on training 132 paid carers in a non randomised clinical study (n = 60) that reported significant reductions in challenging behaviour in people in the target group compared with controls. However, the instrument used to measure the primary outcome does not have established psychometric properties, the study was uncontrolled and included training paid carers (rather than professionals), who are likely to require a different set of skills and knowledge from the outset. The authors estimated that PBS based training may lead to savings of £2 K per person treated.

The central objective of good practice in treating challenging behaviour is maintain individuals who present with such behaviours locally and to avoid transfers to out-of-area facilities [28]. Other guidance advocates a person centred approach encompassing tenets of PBS which leads to environments capable to deal with such behaviour and the implementation of skilled commissioning for care provision [29].

We argue that a rigorous evaluation of the clinical and cost effectiveness of PBS delivered by appropriately trained staff is long overdue. The focus of this project is highly relevant given the widespread implementation of PBS and thus the findings would have significant practice and policy benefits internationally. Moreover, the economic analysis will determine whether the intervention incurs costs beyond and above treatment as usual and any reductions in challenging behaviour are associated with reduced use or resources and improved quality of life.

#### Primary objective

To compare changes in carer reported ratings of challenging behaviour at 12 months in adults with intellectual disability who are treated in teams trained to deliver PBS in addition to TAU with those treated in teams that deliver TAU alone.

#### Secondary objectives

i. To compare the costs of care in each arm.
ii. To examine the impact of the intervention and TAU at 12 months on prescription of psychotropic medication, burden on family and paid carers, service user mental status, and participation in community-based activities compared to TAU alone.
iii. To carry out an exploratory analysis of the impact of the intervention and TAU alone at 12 months on all measures in a sub-sample of participants with autism spectrum disorders (ASD).
iv. To measure the influence on the primary outcome of level of intellectual disability, mental status and ASD status and adaptive behaviour scores.

## Methods

### Trial design

Multicentre cluster researcher-masked randomised controlled trial of manualised PBS-based staff training programme for managing challenging behaviour in adults with intellectual disability. The control group will receive treatment as usual. Treatment as usual will also be available to the participants in the intervention arm. The unit of randomisation will be the community intellectual disability service. A cluster randomised design is considered appropriate in the light of this being an educational intervention aimed at groups of professionals and the service [30].

### Sample size

The primary outcome is the total Aberrant Behaviour Checklist (ABC) score measured repeatedly at six and 12 months following recruitment. The pilot study [15] generated mean baseline ABC scores of 45.4 (SD 26.4). A reduction of 0.45 of a standard deviation on the ABC score in the ABC group compared to the TAU group is considered to be clinically important. Using an analysis of covariance approach based on a correlation of 0.48 between the baseline and post-intervention ABC measurements (as per pilot study, 15) 80 patients per arm will be required to detect a difference of 0.45 SD with 90% power and 5% significance level. Inflating for clustering within the community intellectual disability services, using the formula proposed by Eldridge et al. [31] which accounts for variable cluster sizes and an intracluster correlation of 0.062 (estimated from the pilot study), an average cluster size of 12 (we expect it to be 13, however, allowing for 10% attrition and rounding it up to the nearest integer we have used a cluster size of 12 in our calculations), and a standard deviation for the cluster size of 3, a total of 276 patients will be required. However, this sample size can be reduced as each patient will provide two measurements of ABC score. Using a correlation of 0.6 between the 6 and 12 months post intervention ABC measurements (15,16) and a cluster size of 2, a total of 442 ABC measurements from 221 patients will be required. In performing this calculation we have assumed that there will be no treatment by time period interaction over 12 months supported by the pilot studies [11,12]. To allow for 10% attrition over the 12 month period a total of 246 patients will be recruited to the trial thus requiring 19 clusters. The sample size calculation is based on the program and formulae in STATA version 12.

### Service and participant recruitment

Community intellectual disability services will be recruited across several regions in England which cover urban, semi-rural and rural areas.

We will ask two professionals from psychology, nursing, occupational therapy or speech and language therapy in each of the teams to volunteer to receive PBS training. A further one or two professionals who express interest will be on a waiting list in case of staff changes or drop outs. Each trained professional will have a maximum caseload of eight individuals at any time during the trial, i.e. a maximum of 16 service users per team. They may continue to provide generic input for other service users such as taking part in dysphagia assessments, assessment of capacity or day activities/home environment if practicable. However, as they will be delivering an intensive intervention, their generic workloads will need to be reduced. We anticipate that delivery of the intervention will be about 30% of their time. Once training has been completed, we anticipate that 12–14 service users across sites per month will be taken on for treatment.

### Randomisation

Once the services have been recruited, randomisation will be performed using a web based randomization system which uses random permuted blocks on 1:1 allocation. Although originally we had discounted minimisation given the lack of good evidence for the impact of various variables on patient outcomes, given the variation in cluster sizes we will use the following data in our randomisation planning: team size (number of full time equivalent staff), in or out of London, number of service users registered per team. We have calculated the ratio of staff:patient in and out of London teams and in order to account for the variation we will stratify the teams by a binary factor to indicate whether a team is below or above a median ratio. Randomisation will take place after the participants have been screened for eligibility and consented to the trial. Baseline data collection will follow.

### Masking

Research assistants but not teams will be blind to group allocation of the service. They will be asked to retain a diary of their estimation of which treatment the participant is allocated to and to report occasions where this has been disclosed to them in the course of their work. Unmasking will not occur until after the study endpoint and once the database is locked. Finally we will compare guesses about participant allocation between trial arms in order to determine any loss of blinding and potential bias and we will adjust for any bias detected.

### Possible sources of bias

One potential source of bias is transfer of trained staff between trained and control teams during the trials. We have examined the turnover of health staff in a number of services which have expressed interest in participating and this is well below 13% which is considered very low based on National Health Service performance data. Furthermore contamination between study arms is unlikely given the more intensive nature of the intervention. However, PBS principles are being taught widely therefore, some knowledge of PBS will be unavoidable. We will collect information from all participating teams on the level of knowledge and training the staff may have had in delivering any aspects of PBS. Another potential source of bias may derive from variation in patient characteristics between teams. However, we do not anticipate substantial variation in this respect as the teams will cover diverse geographical areas thus reducing the possibility of bias.

Selection bias resulting from recruiting participants after cluster allocation has been revealed will be avoided by completing recruitment and screening assessment prior to randomisation.

### Inclusion criteria

- Service users: Eligible to receive care from intellectual disability services; mild to severe intellectual disability; aged 18 years and over; total ABC score of at least 15 (indicates a degree of challenging behaviour occurring at least weekly including verbal or physical aggression, hyperactivity, refusal to attend activities, non responsiveness that requires professional input).
- Service: Willing to participate; availability of at least two staff members willing to train; written agreement by the service manager to participate.

### Exclusion criteria

- Service users: primary clinical diagnosis of personality disorder or substance misuse; relapse in pre-existing mental disorder; decision by clinical team that a referral to the study would be inappropriate, e.g. there is an open complaint investigation.
- Service: there are no team members willing to train; the service has already received and implements PBS for their service users.

### Interventions

#### PBS based staff training (in addition to treatment as usual)

The training, which will be supported by a treatment manual will comprise the following sections:

- Functional Behavioural Assessment and formulation skills

- Brief Behavioural Assessment Tool for brief functional analyses

b) Primary Prevention
c) Secondary Prevention and Reactive Strategies
d) Periodic Service Review and Problem Solving

- Developing individualised periodic service reviews
- Trouble shooting

The training will take place on six days spread over 15 weeks. Section (a) will be delivered over a two day workshop and sections (b) to (d) over another two day workshop six weeks later. Participating proflessionals are expected to begin undertaking functional assessments of service users’ between the two workshops. The final two-day workshop eight weeks later will include discussion on how to effectively implement behavioural plans and provide problem solving strategies.

Post-workshop mentoring arrangements have been arranged.

While mentors will take all practical steps to ensure appropriate contacts are put in place, responsibility for making best use of supervision rests with participants. Throughout the training and mentoring, participants will be reminded that the present training model is aimed at intervention for individuals who do not show the most complex challenging behaviours; intervention for such individuals will require more intensive and skilled intervention. Clinical responsibility for each case and for responding to any emergencies remains with the local services. When a service user presents in a clinical emergency, the clinical team will manage the emergency following which the treatment will be resumed as appropriate.

At the end of treatment, the service users and their paid or family carers will receive a report-in accessible format for the service users- that describes the main gains from treatment and strategies to use if problems arise again.

One day training seminar on PBS will be offered to the teams allocated to the control arm at the end of the study.

#### Treatment as usual (TAU)

Most community intellectual disability services provide a range of health interventions that include but are not limited to psychiatric assessment and management, nursing support, psychology, speech and language therapy, occupational therapy and counselling. There may be some variation in resources but service users with challenging behaviour are likely to receive a range of broadly defined behavioural, psychosocial and pharmacological interventions. The latter may be influenced by or broadly based on treatment guidelines published by the British Psychological Society and the Royal College of Psychiatrists [32].

### Frequency and duration of follow up

**Figure 1:**
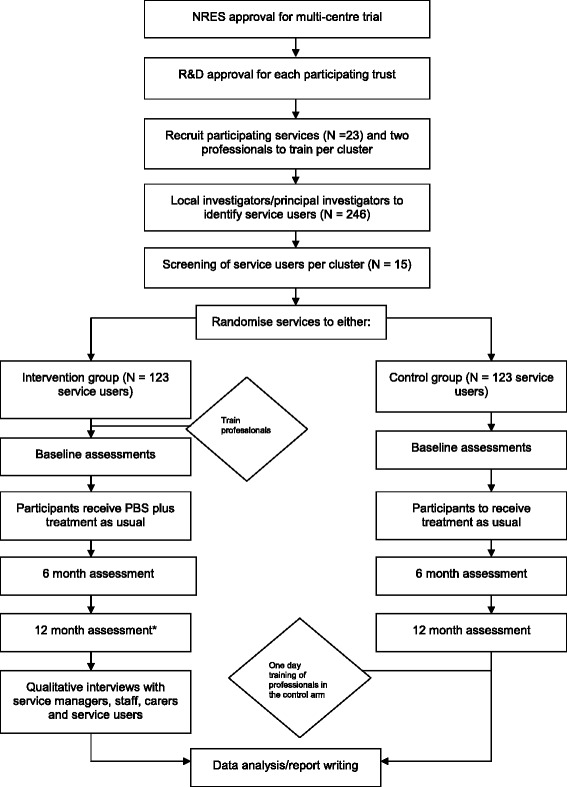
**Trial schema.**

All participants will be followed up at six and 12 months after randomisation. The flow of the study can be seen in Figure 1.

### Ethical issues, research governance and consent

The study has received ethical approval by the NRES Committee London - Harrow (reference 12/LO/1378). All materials relevant to the study were made into easy read with the assistance of the service users reference group. Research and Development permissions have also been obtained for the participating teams. Details can be found in the Additional file 1: Table S1.

Written consent to contact patients and collect data for the purpose of the study will be obtained from the service users where possible, and family and paid carers. We will identify a consultee where one is available, or nominate one for those service users who lack capacity as per Mental Capacity Act 2005 [33]. Our previous work suggests that PBS is well received, of potential benefit and does no harm. As with any trial which includes TAU, participants may have a preference for the active treatment. However, it remains to be determined whether training staff in PBS is effective. Where continued participation would be inappropriate, e.g. admission to hospital under detention of the MHA 1983, participants will be withdrawn from the study. The service user’s General Practitioner will be notified of the service user’s participation in the trial. All the research team members will follow the required risk assessment procedures including guidelines for risk management and safeguarding processes.

### Outcome measures and instruments

#### Quantitative assessments

We will collect data on demographic characteristics (gender, age, ethnicity), level of intellectual disability and adaptive behaviour at baseline. The latter two will be assessed by the Wechsler Abbreviated Scale of Intelligence (WASI) [34] and the short version of the Adaptive Behaviour Scale [35] respectively. Cause of learning disability will be recorded if known.

#### Primary outcome measure

Changes in challenging behaviour as measured by the Aberrant Behaviour Checklist [36,37] over 12 months. The instrument has been widely used for monitoring changes in behaviour in people with intellectual disability following treatment and has demonstrated acceptable reliability and validity. The ABC scores can be separated into five different factors comprising (I) Irritability, Agitation, Crying (15 items), (II) Lethargy, Social Withdrawal (16 items), (III) Stereotypic Behaviour (7 items), (IV) Hyperactivity, Non-compliance (16 items), and (V) Inappropriate Speech (4 items). Each domain is rated on a four point scale (0–3). A total score can be obtained by adding up all domain scores. It will be completed by the person’s paid or family carer at all time points.

#### Secondary outcome measures

Mental health and autism spectrum disorders will be assessed by the mini version of the Psychopathology Assessment Scale for Adults with Developmental Disability (mini PASADD) [38]. It is administered by trained professionals to family and/or paid carers and to the service user where possible. The instrument comprises 86 psychiatric symptoms with threshold scores for the following psychiatric disorders: Depressive Disorder, Anxiety Disorder, Hypomania/Mania or Expansive mood, Obsessive Compulsive disorder, Psychosis, Dementia or Unspecified Disorder and a screen for pervasive developmental disorder. The latter will be administered once at baseline.

EuroQol Youth (EQ-5D-Y) will be used to calculate Quality Adjusted Life Years (QALYs) in line with accepted guidance [39]. The EQ-5D-Y is a 5 domain (usual activity, self-care, mobility, pain and anxiety/depression), 3 level (no problems, some problems and extreme problems) questionnaire. It will be administered at all time points.

Community participation will be assessed with the Guernsey Community Participation and Leisure Activities Scale (GCPLAS) [40]. It was developed to monitor the impact of interventions on the service user’s daily living. It contains six categories of activity that refer to 49 operationally defined contacts. The frequency of participation over the previous six month period is rated on a five point scale. It will be administered at all time points.

Family carer burden will be measured by the Uplift/Burden Scale [41], a 23-item scale that has six uplift and 17 burden items. The scale has previously been used in this population (6). It will be administered at all time points.

Family carer psychiatric morbidity will be measured by the GHQ12 [42].

Paid carer burden will be measured by the Caregiving Difficulty Scale-Intellectual Disability (CDS-ID) [43]. It is adapted from an existing scale and measures subjective burden. It will be administered at all time points.

Costs of care will be collected using a modified version of Client Services Receipt Inventory (CSRI) for people with intellectual disability [44]. It will be administered at all time points for the preceding 6 months.

Use and/or change of all medications will be recorded during the study period.

### Qualitative component

We will carry out audiotaped interviews of i) a purposive sample of 15 staff that have undergone training in PBS to explore their views on the training and challenges they faced by taking part in the study; ii) all service managers in order to explore their viewpoint in terms of the impact of the training on the service and the practices that may underpin service response to challenging behaviour; iii) up to 15 paid and 15 family carers for their views and experiences of having been allocated to the intervention arm; iv) up to 15 service users who have experienced the intervention. We will develop a semi-structured interview schedule with the help of both paid and family carers and service users. It will be applied flexibly and may be modified in order to be responsive to emergent themes. If the intervention is shown to be effective, this data will inform the scaling up of the intervention across community intellectual disability services in the UK. If it is ineffective, the data may give some indication of where and why it failed.

### Process evaluation

We will develop a checklist specifically for the study to monitor treatment fidelity. It will ask professionals delivering the intervention to rate whether or not essential tasks were carried out in sessions (adherence) on a yes/no rating. It will also contain a section for comment and to rate whether the treatment goals were achieved by the final session. Furthermore, the list of topics for discussion in supervision sessions with trainers will be available as a record of any issues arising during the trial. Samples of behavioural plans will be checked by the training team as well as an independent advisor to ensure that the quality of the assessments is maintained throughout the trial. Finally we will collect information regarding the resource implication for delivering the intervention in terms of staff time spent in training, supervision or travelling.

### Statistical analysis

We shall follow CONSORT guidelines for the analysis of cluster randomised trials and reporting of findings (http://www.consort-statement.org/).

The baseline characteristics of the PBS and TAU groups will be summarised using means, standard deviation and proportions as appropriate. A three level regression model adjusting for baseline ABC measurements, time period and effects of clustering by services and accounting for repeated measures within subjects will be used for the primary analysis. Similar analyses will be conducted for the secondary outcomes using appropriate regression models depending on the type of outcome. Results from all secondary analyses will be presented as estimates with confidence intervals and treated as exploratory. If we encounter missing data, bias due to missing data will be investigated initially by comparing the characteristics of the trial participants with complete follow-up measurements and those who have incomplete follow-up or no outcome data, descriptively. If differences are observed in the participants’ characteristics in the descriptive analysis, factors which are likely to be associated with the outcome will be included in a multilevel logistic regression analysis (with missing yes or no as outcome) to identify predictors of missing data. If predictors associated with both missing data and the outcomes are found, these will be included in the multilevel models examining the effects of intervention on the outcomes [45]. A sensitivity analysis will also be carried out to examine the effect of PBS on ABC score after adjusting for area deprivation and the service staff to patient ratio if appropriate. Multiple imputation may be used as part of the sensitivity analysis accounting for the clustered nature of the data if considered appropriate [46-48]. As part of the sensitivity analysis we will also examine intervention by time period interaction. All analyses will be carried out on an intention to treat basis. A detailed statistical analysis plan will be agreed by the Trial Steering Committee prior to the analysis of unblinded data.

### Health economic evaluation

Analyses will be conducted from a health services perspective and from a societal perspective. The cost-effectiveness measure will be the incremental cost per QALY gained of PBS versus treatment as usual. This will be calculated as the mean cost difference between PBS and treatment as usual divided by the mean QALY difference to give the incremental cost-effectiveness ratio (ICER). This will allow for resource allocation decisions across different health services. However, as there is no evidence of the relationship between challenging behaviour and QALYs, we will also calculate the cost per point change in the primary outcome measure, (ABC). Unit costs will be taken from standard published sources such as the Office for National Statistics and Personal Social Services Research Unit (PSSRU). The cost of PBS will also include staff resources, supervision and training. Additional information on hospital admissions and out-of-area transfers will be collected to ensure costings of the secondary care component for both trial arms are robust. Predetermined values will be used [49,50] to calculate the area under the curve using time-weighted averages of utility scores collected at baseline and each follow up point. As baseline utility scores are not controlled for prior to randomisation, utility scores may differ between trial arms at baseline. Thus, regression analysis will be used to control for differences in baseline utility scores. We will use non-parametric methods for calculating confidence intervals around the ICER based on bootstrapped estimates of the mean cost and QALY differences. The bootstrap replications will also be used to construct a cost-effectiveness [51] acceptability curve, which will show the probability that PBS is cost-effective compared to standard care at 12 months for different values of the willingness of the NHS to pay for an additional QALY. We will also subject the results to extensive deterministic (one-, two- and multi-way) sensitivity analyses.

### Qualitative analysis

The audiotaped interviews will be transcribed by the study administrator and the research assistants will import the transcripts onto suitable software [52] that will be used to manage the data. The thematic analysis of the nested qualitative study will be guided by the framework approach [53]. Codes will be assigned to segments of interview data according to agreed labels. Once codes are applied to interview data, the outcome will be checked by another member of the research group to monitor the face validity of the assigned cases and add further codes. Higher level themes will be suggested in discussions with the research group. Furthermore, we will perform further validation of the material by presenting the participants with a summary of the findings in order to establish that the account we have given is clear and acceptable to the respondents.

### Patient and carer involvement

A patient reference group (Camden Advocacy) will support the project and will assist with the research governance requirements including input to easy read versions of trial materials such as information and consent forms, piloting of instruments and qualitative interview topic guides with reference to people with intellectual disability and dissemination. The Challenging Behaviour Foundation, an independent charity of family carers of people with intellectual disability and challenging behaviour were involved in the development of the study and have taken on the role of champions for recruitment, reviewing study related information, the qualitative interviews topic guides and final dissemination of the findings.

## Discussion

Large scale comparative evaluations of training in PBS are lacking. It is anticipate that the trial findings will provide important information about its clinical and costs effectiveness as well as its delivery in ordinary practice. This is of particular interest to both professionals and clinicians as PBS has been embraced as the intervention of choice for managing challenging behaviour by a wide spectrum of stakeholders.

## Additional file

Additional file 1: Table S1. R & D departments and corresponding sites in the participating NHS Trusts.
