# Supplementary material for: Clinical and cost effectiveness of staff training in Positive Behaviour Support (PBS) for treating challenging behaviour in adults with intellectual disability: a cluster randomised controlled trial
Source: BMC Psychiatry. 2014 Aug 3;14:219. doi: 10.1186/s12888-014-0219-6 (PMC4149205; doi:10.1186/s12888-014-0219-6)
Supplement: Additional file 1: Table S1. — R & D departments and corresponding sites in the participating NHS Trusts. [file 12888_2014_219_MOESM1_ESM.docx]

**Supplementary table 1: R&D departments and corresponding sites in the participating NHS Trusts**

| **R&D approval** | **Team** |
| --- | --- |
| Camden and Islington NHS Foundation Trust (NoCLoR) | *Camden Learning Disability Service* |
|  | *Islington Learning Disabilities Partnership* |
| Barts Health NHS Trust & East London NHS Foundation Trust | *Tower Hamlets Community Learning Disability Service* |
| Homerton University Hospital NHS Foundation Trust | *Hackney Learning Disabilities Service* |
| Barnet, Enfield and Haringey Mental Health NHS Trust (NoCLoR) | *Barnet Learning Disabilities Service* |
| Central and North West London NHS Foundation Trust (NoCLoR) | *Enfield Integrated Learning Disabilities Service* |
| North East London NHS Foundation Trust | *Barking & Dagenham Community Learning Disability Service* |
|  | *Havering Community Learning Disability Service* |
|  | *Waltham Forest Community Learning Disability Team* |
| Oxleas NHS Foundation Trust | *Bexley Learning Disability Team and Greenwich Community Learning Disability Team* |
| South West London & St George’s Mental Health NHS Trust | *Wandsworth Community Learning Disability Team* |
| Leicestershire Partnership NHS Trust | *Leicester City (East) Community Learning Disability Team* |
|  | *Leicester City (West) Community Learning Disability Team* |
|  | *Charnwood Community Learning Disability Team* |
|  | *Coalville & Hinckley Community Learning Disability Team* |
|  | *Market Harborough, Oadby and Wigston Community Learning Disability Team* |
| Kent & Medway NHS and Social Care Partnership Trust via RM&G Consortium for Kent and Medway | *Dartford, Gravesend & Swanley Mental Health of Learning Disability Service* |
|  | *Medway Mental Health of Learning Disability Service* |
| Surrey and Borders Partnership NHS Foundation Trust | *East Surrey Community Team for People with Learning Disabilities* |
|  | *South West Surrey, NE Hants & North West Surrey Community Team for People with Learning Disabilities* |
|  | *Mid Surrey Community Team for People with Learning Disabilities* |
| Coventry & Warwickshire Partnership NHS Trust | *Coventry, South Warwickshire & Rugby Community Learning Disabilities Teams* |
| Bradford District NHS Care Trust | *Bradford Learning Disabilities Service* |
